# Antagonism of Remedies

**Published:** 1880-12

**Authors:** 


### ANTAGONISM OF REMEDIES.

We clip the following extract on the antagonism of morphia and the alkaloids of tea, coffee, and other remedies, from the fourth lecture in the Cartright series, delivered by Dr. Robert Bartholow, in Philadelphia, and published in the *Medical Record* of December 11, 1880:

One of the most interesting researches undertaken by the committee of the British Medical Association, of which Dr. Bennett was chairman, was that in regard to the supposed antagonism between morphia on the one hand and the alkaloids of coca, tea, coffee, and guarana on the other. It was found that the physiological action of these alkaloids was practically identical; their effects being "cerebral excitement, succeeded by coma, when the quantity is large; loss of sensibility, which is partial when the dose is large; tetanic spasms and convulsions; and paralysis of the posterior columns of the spinal cord and the peripheral sensory nerves, leaving the anterior columns and the motor nerves unaffected. At first they increase and finally they diminish the force and frequency of the cardiac contractions, and first irritate and then paralyze the vasomotor nerves." It will be seen, therefore, that some of these actions are promoted, and others opposed by morphia. The researches of the committee demonstrate that there is some foundation for the popular idea that strong decoctions of tea and coffee are of service in opium-poisoning. Thus, they found that theine was antagonistic to miconate of morphia, and that the action of one so far modified that of the other as to avert death after a fatal dose of either.

MORPHIA AND CHLOROFORM.

As paralysis of the heart or of the respiration, or possibly by the simultaneous depression of both functions, is the mode of death from chloroform and other anæsthetics, it is certainly very desirable that we should have an agent which will antagonize and prevent this fatal tendency. In the subcutaneous injection of morphia I am entirely convinced that we do possess such an agent; and it is a matter of great surprise to me that surgeons have not more generally availed themselves of the indisputable advantages of mixed anæsthesia. It was about the same time that Claude Bernard and Nussbaum demonstrated the great utility of the method of inducing anæsthesia by the subcutaneous injection of morphia combined with the inhalation of chloroform—Bernard administering the morphia a few minutes before commencing the inhalation, and Nussbaum not until the latter was well under way.

Morphia and chloroform act on the same cellular elements of the brain, and agree in the production of anæsthesia, but they are opposed in their action on other structures and organs—an opposition which renders their combined use safer. When morphia is injected before the inhalation of the anæsthetic is begun (which is the preferable method on account of the manner in which it facilitates the latter), the irritability of the bronchial mucous membrane is so far overcome as to permit the inhalation to proceed quietly; the stage of excitement is prevented, and consequently the danger from asphyxia which sometimes accompanies this; the nausea and vomiting are also obviated, and the anæsthetic effect is prolonged without the aid of further inhalation. In addition, the nausea and vomiting, after-pain and depression which follow the use of anæsthetics, as well as the dangerous syncope which sometimes results, can be prevented to a great extent by this method. If the morphia and the chloroform inhalation be carefully and properly combined, it is possible to produce anæsthesia without loss of consciousness, a point in regard to which Bossis says in his thesis on this subject: "There may be obtained in man, with a little attention, by the combined action of chloroform and morphia, a state of complete insensibility to pain, with preservation, to a partial extent, of the intelligence, tactile sensibility, auditory and visual, and of the voluntary movements. From a practical point of view, the analgesia obtained by the combined action differs completely from the demianæsthesia caused by the employment of chloroform or ether singly, in that it is not preceded or accompanied by a period of hyperæsthesia with violent excitement, and the tendency to exaggerated reflex arrest of the heart and after-syncope."

From the practical experience thus far accumulated there can be no doubt that morphia, used after the method of Bernard, greatly facilitates the induction of anæsthesia and materially diminishes its dangers. I have maintained that for this purpose atropia in combination with morphia should be preferred to morphia alone, on account of the greater stimulating effect thus produced upon the cardiac and respiratory centres. It might, perhaps, be supposed that atropia alone would be better than morphia; but it must not be forgotten that stimulation is inevitably followed by reaction, and morphia has a power of continued support which atropia does not possess. When administered together under the circumstances, the evil effects of both are antagonized, and the power of both to support the heart and respiration utilized. The quantity of morphia employed should rarely exceed one-fourth of a grain, and of atropia one-hundredth of a grain.

(Having spoken of the effect of strychnia as a stimulant of the respiratory function, the lecturer proceeded as follows:)

An opposition of actions has been determined to exist between strychinia and nitrite of amyl. These substances act antagonistically on the nervous system of animal life and on the sympathetic system. Nitrite of amyl suspends the reflex function of the spinal cord, and causes paralysis of the muscular system, death ensuing from paralysis of the respiratory muscles. Its most characteristic effects are those upon the heart and arterial system; the arterial tension being depressed to the lowest point, while the action of the heart is necessarily very greatly increased on account of the enormous dilatation of the peripheral vessels produced. These effects being the opposite of those caused by strychnia, an antagonism may be presumed from a physiological standpoint, and the experimental rasearches of Dr. Gray, of Glasgow, strongly support this view.

(The hour having now expired, Dr. Bartholow was obliged to omit a portion of the lecture, and concluded in the following words:)

With the antagonisms discussed in this lecture rather hastily, as the subject matter of the course will permit no fuller treatment, I close this part of the subject, or the antagonisms between medicines. I have yet to discuss, in the remaining lectures, that large and important practical subject—the antagonism between remedies and diseases.

In my next lecture, I will, therefore, begin the consideration of this topic, which will not only enforce the lessons derived from the study of the physiological antagonisms between medicines, but will, I hope, demonstrate a path which we may surely follow in the treatment of many diseases.

